# Functional Peptides from SARS-CoV-2 Binding with Cell Membrane: From Molecular Dynamics Simulations to Cell Demonstration

**DOI:** 10.3390/cells11111738

**Published:** 2022-05-25

**Authors:** Yun Hao, Rongrong Wu, Fenghua Wang, Liwei Zhang, Zengkai Wang, Xiaolu Song, Lei Liu

**Affiliations:** Institute of Advanced Materials, Jiangsu University, Zhenjiang 212013, China; haoyunmxx@163.com (Y.H.); 15617396130@163.com (R.W.); fenghua_hc@163.com (F.W.); lwzhangujs@163.com (L.Z.); wzk0319@163.com (Z.W.); songxl@ujs.edu.cn (X.S.)

**Keywords:** SARS-CoV-2, functional peptides, lipid membrane, interaction, new materials based on peptides

## Abstract

Herein, we have verified the interaction between the functional peptides from the SARS-CoV-2 and cell membrane, and we further proved that peptides exhibit little membrane disruption. The specific amino acids (Lys, Ile, Glu, Asn, Gln, etc.) with charge or hydrophobic residues play a significant role during the functional-peptide binding to membrane. The findings could provide the hints related to viral infection and also might pave the way for development of new materials based on peptides with membrane-binding activity, which would enable functional peptides further as peptide adjuvants, in order to help deliver the cancer drug into tumor cells for the efficient tumor therapy.

## 1. Introduction

The virus displays a strong capacity to invade cells, leading to infection and transmission [1,2,3]. For instance, Spike glycoprotein (S protein) in the severe acute respiratory syndrome coronavirus 2 (SARS-CoV-2) plays a significant role in the virus entering into the host cells, leading to high infectivity [4,5,6]. Although the key mediation of virus invasion is considered as the binding of the receptor-binding domain (RBD) and receptor angiotensin-converting enzyme 2 (ACE2), the fusion peptide in the S2 fragment exposed after the binding of the RBD-ACE2 still plays a significant role in the insertion into the host membrane to perform membrane fusion between virus and host cell [7,8]. Membrane fusion is a crucial step for successful entry and infection of enveloped viruses, leading to the transfer of viral genetic materials into the host cell [9,10]. However, the molecular insight of the peptide interactions with the host membrane and in which the domain of peptides plays a key role in membrane fusion is currently attracting limited but highly anticipated attention.

A critical group of viral fusion proteins [11,12,13] in the coronavirus family including the SARS virus, MERS virus, and influenza virus, have been explored to prove their ability to directly interact with and insert themselves into the host cell membrane, thereby initiating the fusion process [14,15]. For instance, fusion protein in influenza virus hemagglutinin has a pronounced effect on the membrane, significantly reducing the bilayer thickness in the surrounding region and disturbing the order of the nearby lipids, which facilitates the fusion process [13]. In the case of the isolated fusion protein of the MERS virus spike protein, it was verified that the peptide could increase the size and deform the shape of giant unilamellar vesicles (GUVs) [12]. Each fusion protein displayed the diverse interaction way with lipids, exhibiting the significant role of mediating the virus invasion. 

Therefore, the fusion peptides of the virus or peptides from the virus with similar effect of membrane binding are valuable to explore systematically, due to the significance for understanding the virus invasion or proposal of new functional peptides as biomaterials to bind cells. At present, many attempts have been made to explore the key region and activity of fusion peptides of SARS-CoV-2; however, most of the explored peptides are long-sequence, exhibiting membrane-fusion properties [16,17,18,19]. We extracted two functional-peptide sequences from the S protein of SARS-CoV-2, which might have similar membrane-binding activity, and we defined them as the functional peptides, FP-1 and FP-2. Thus, we thought that the isolated peptide sequence could be used as a model to study the interaction between this region and the lipid membrane. Therefore, in this work, we explored the interactions of the two functional peptides, (FP-1, IYKTPPIKDFGGFNFSQIL [20] and FP-2, IAVEQDKNTQEVFAQVKQI [21]) of SARS-CoV-2 with cell membrane combined with the experiments and molecular dynamic simulations [22]. The specific amino acids (Lys, Ile, Glu, Asn, Gln, etc.) with charge or hydrophobic residues were determined to play a significant role during the functional-peptide binding to the membrane, which could be key to our understanding of critical steps related to viral infection and also might pave the way for development of new materials based on peptides with membrane-binding activity. The findings would enable functional peptides further as peptide adjuvants in order to help deliver the cancer drug into tumor cells for efficient tumor therapy.

## 2. Materials and Methods

### 2.1. Experimental Details

#### 2.1.1. Materials

All of the chemicals used were of analytical grade. The functional peptides FP-1 and FP-2, and their peptide conjugates, Fluorescein-5-isothiocyanate (FITC), FP-1-FITC and FP-2-FITC were synthesized by Guoping Pharmaceuticals (Hefei, China) with 98% purity. 1,1,1,3,3,3-Hexafluoro-2-propanol (HFIP) was purchased from Tokyo Chemical Industry (Tokyo, Japan). Cell Counting Kit (CCK8) was purchased from Solar bio (Beijing, China). Phosphatidylcholine DPPC (1, 2-dihexadecanoyl-sn-glycero-3-phosphocholine) was purchased from Avanti Polar Lipids Co., Ltd (Birmingham, AL, USA). 2-(4-Amidinophenyl)-6-indolecarbamidine dihydrochloride (DAPI) and cell-membrane orange-red fluorescent probe (DIL) staining solution were purchased from Beyotime (Shanghai, China). The mouse fibroblast cell line (L929) was obtained from normal loose subcutaneous connective tissue of mice, and the cell line L was cloned. The L929 cell line was provided by Suzhou Bena Tronlink Biotechnology Co., Ltd (Suzhou, China).

#### 2.1.2. Preparation and Characterization of Functional Peptides

To prevent any preaggregation, the peptides were firstly dissolved in HFIP. An appropriate amount of the dissolved peptide solution was dried in a vacuum oven (PTC-348W1, JASCO Co. Dongjing, Japan) and then dissolved with phosphate buffer (PBS, pH 7.4) to obtain a peptide solution (66 μΜ). Circular dichroism (CD) analysis was performed to investigate the secondary structure of the functional peptides and their conjugates (JASCO, Hachioji City, Japan). CD of the secondary structure of FP-1 and FP-2 in solution. All the experiments were performed in triplicate.

#### 2.1.3. Preparation of DPPC Liposomes

DPPC powder were firstly dissolved in chloroform and dried in vacuum oven overnight (Jinghong Co., Ltd. Shanghai, China). The DPPC film was hydrated with the phosphate buffer solution (PBS, pH 7.4) and sonicated for 30 min to obtain a DPPC solution (10 mM). The resulting DPPC solution was alternately subjected to 10 freeze–thaw cycles in liquid nitrogen and a 51 °C water bath, and then extruded 21 times through a 1 μm polycarbonate membrane using a liposome extruder (Avanti Polar Lipids, Birmingham, AL, USA). The calcein solution (5 mM) was added into the prepared DPPC solution (10 mM), and the mixture solution was alternately subjected to 10 freeze–thaw cycles, followed by ultrafiltration. Then, the calcein-encapsulated liposomes were obtained.

#### 2.1.4. Dynamic Light Scattering (DLS)

The DPPC liposomes obtained by extrusion were characterized by laser particle-size analyzer (MS3000) to study the particle-size distribution. The starting concentration of DPPC liposomes was 1 mM. Experiments were performed in phosphate buffer saline pH 7.4 using a Zetasizer nano zs system from Malvern Instruments, and the sample was placed in a colorimetric dish for analysis and testing 3 times. The temperature was maintained at 25 °C throughout the experiment. 

#### 2.1.5. Calcein-Release Assay

To prove the interaction between the functional peptides and liposome (DPPC), calcein-release assay was performed by microplate reader (Biotek Synergy H1, Winooski, VT, USA). The prepared functional peptides at different concentrations (molar concentration ratio of peptide: lipids were 1:100, 1:50, 1:10, which were 10 µM, 20 µM, 100 µM.) were pipetted in diluted calcein-encapsulated liposomes (1 mM) with the ratio of 1:1 (*v/v*). The negative control was measured without peptide, and 1% (*v/v*) Triton X-100 was added into calcein-encapsulated liposomes solution as a positive control. Then, the addition of the peptides to the liposome solution was recorded by the fluorescence spectrum after 2 h. The measurement of fluorescence spectra was recorded between 520–620 nm with an excitation wavelength of 470 nm at room temperature [23].

#### 2.1.6. Liposome Localization of the Functional Peptides

The functional-peptide conjugates (FP-1-FITC, 100 µM) were added into the pure DPPC liposomes (1 mM) and incubated for 2 h, and then observed by laser confocal system (Leica TCS SP5, Heidelberg, Germany).

#### 2.1.7. Cell-Membrane-Localization Assay

The suspension of the mouse fibroblast cell line (L929) was transferred to a 48-well plate. After 24 h of incubation, the plate was washed with PBS buffer to remove any nonadherent cells. The functional peptides were added to the well plate and incubated for 4 h, and the cells were washed with PBS buffer to remove any nonspecifically bound functional peptide. The obtained cells were fixed with a paraformaldehyde fixative for 30 min, washed with PBS, and then the nuclear-specific dye DAPI and membrane-specific dye DIL were added to interact with the cells for 15 min and washed with PBS. Finally, they were observed under a fluorescence microscope at room temperature [24,25].

#### 2.1.8. Cytotoxicity Measurement

The mouse fibroblast cell line (L929) was seeded at a density of 5 × 103 cells per well onto a 96-well plate and incubated for 24 h at 37 °C in an incubator with 5% of CO_2_. The functional-peptide solution (10 µL) at various concentrations (20, 40, 100, 200 µM) was mixed with 90 µL fresh culture medium in a 96-well plate. After 24 h incubation, the cytotoxicity was assayed using CCK8. The cell without incubation with peptides was used as a control. All the experiments were performed in six replicates.

#### 2.1.9. Statistical Analysis

All quantitative data were presented as mean ± standard deviation (S.D.) with no fewer than three replicates for each experimental condition. Statistical analyses were mainly performed using one-way analysis of variance (ANOVA) followed by Tukey’s test. Two-way analysis of variance was only used when comparisons were made with two or more interconnected variables. Differences between two groups were considered significant when the *p*-value was less than 0.05.

### 2.2. Simulation Details

#### 2.2.1. Set Up of the Functional Peptide and Membrane Systems

Two presumptive functional peptides were separated from the SARS-CoV-2 S protein (Protein Data Bank, PDB ID: 6VXX). We are interested in analyzing internal molecular mechanisms about the interactions of two presumptive functional peptides with membranes models. Four different lipid-membrane models were adopted to simulate the biological membrane-fusion environment, which were DPPC (1,2-dipalmitoyl-sn-glycero-3-phosphocholine) and POPC (1-palmitoyl-2-oleoyl-sn-glycero-3-phosphocholine). POPG (1-palmitoyl-2-oleoyl-sn-glycero-3-phosphoglycerol) DMPC (1,2-dimyristoyl-sn-glycero-3-phosphocholine). These lipid-membrane systems present very different physicochemical characteristics. At physiological pH, PC lipid membrane is in zwitterionic form, having no net charge, while POPG membrane is in anionic form, carrying a negative charge per POPG molecule. This difference is interesting because it allows us to analyze the effect of the charge on the interactions with the peptides. Additionally, from a biological point of view, we can consider PC lipid bilayers as the membrane model of mammalian cells and the POPG lipid bilayers as the bacterial-membrane model [26].

#### 2.2.2. All-Atom Molecular Dynamics Simulations

For the pure lipid-membrane system, there were 128 lipid molecules, 6102 water molecules, and 0.15 M NaCl ions added in the box with a size of 6.3 × 6.3 × 9.0 nm^3^. Four boxes consisting of a lipid bilayer of DMPC, DPPC, POPC, or POPG were arranged. After 40 ns equilibrations, two presumptive functional peptides were separately introduced into these four different lipid-membrane systems. The functional peptide was initially placed at 1.2 nm above the lipid membrane vertically, and it was oriented with the first principal axis (the larger) parallel to the lipid bilayer surface, and each system was simulated for 500 ns.

All simulations were performed using the program GROMACS 2020. The GROMOS96 53a6 force field and the SPC water model were chosen for the lipid membrane and water molecules, respectively. Periodic boundary conditions of these systems were set for all directions. The NPT (T = 298 K and P = 1 bar) ensemble was applied with the pressure regulated using a Berendsen barostat and temperature-controlled using a velocity-rescale thermostat. The calculation of long-range electrostatic interactions was managed by the Particle Mesh Ewald (PME) method. The cutoff distances of the van der Waals (vdW) interactions and electrostatic interactions were set to be 1.2 nm. Snapshots were rendered by using the visual molecular dynamics (VMD) program [27].

#### 2.2.3. Lateral Diffusion Coefficients

Lateral diffusion coefficients for long-time stands were averaged over lipid molecules and calculated from the mean-square displacement (MSD) of the center of mass 〈(r_i_(t) − r_i_(0))^2^〉 in two dimensions, where 〈〉 refers to the average taken over all starting times τ and r(t) to the position of the center of mass at time t. The lateral diffusion coefficient is then given by the Einstein relation MSD = 4D·t in two dimensions for long times t and is averaged over 128 lipid molecules. In order to obtain the long-range diffusion coefficients, the MSD were fitted between 50 and 450 ns. Calculations have been performed with the g_msd program of the GROMACS suite. Lateral lipid-diffusion coefficients were subsequently extracted in the presence of each functional-peptide variant (D_mem+functional peptide_). In order to measure diffusion coefficients in the absence of the functional peptide (D_mem_), independently, these pure lipid-membrane systems of DMPC, DPPC, POPC, or POPG were also simulated for 500 ns. We calculated equivalent “interaction ratios” from the simulations of each construct (D_mem+functional peptide_/D_mem_) to evaluate the interaction between the lipid membrane and the functional peptide.

#### 2.2.4. Umbrella Sampling

Potentials of mean force (PMF) were calculated using the umbrella sampling algorithm for the most dominant cluster. Starting with an initial position of a peptide in the bilayer center (Z = ±0.0 nm), the fusion peptide was pulled away from the bilayer surface along the direction of the bilayer normal (Z axis). A harmonic potential with a force constant of 1000 kJ mol^−1^ nm^−2^ was applied between the center of mass (COM) of the functional peptide and a reference position along the Z axis determined with respect to the COM of the membrane. The motion of the peptide in the X-Y plane was not restrained. All trajectories were used to unbiased umbrella samplings using the weighted histogram analysis method (WHAM). The data were subsequently unbiased to yield the associated PMF profile. A typical PMF A(ζ) has the following characteristics: it lowers with increasing COM distance of the Cα atoms between the fusion peptides and lipid layers (Z axis) until the optimum distance is reached at the minimum PMF. After that, the PMF increases until the fusion peptides and the lipid layers cease to influence each other and the PMF reaches a plateau. These 24 windows were equilibrated for 10 ns and then used as starting configurations for umbrella sampling simulations. Each window was simulated for 60 ns, leading to a total of 1440 ns for 24 windows of each peptide–bilayer system [28].

#### 2.2.5. Curvature Analysis of Lipid Membrane

In order to obtain the changes in the morphology of four lipid membranes, we used MATLAB software to compare the morphology of the lipid membranes in the presence and absence of the functional peptides in the X-Z plane at the last simulation time (t = 500 ns) for each system. We extracted the X, Y, and Z axis of phosphorus atoms on the superior lobule of the lipid layers. We used our own script to make the morphology of phospholipid in MATLAB software, and calculated the curvature of phospholipid in X-Z plane with and without fusion peptides for numerical analysis.

## 3. Results and Discussion

**Figure 1:**
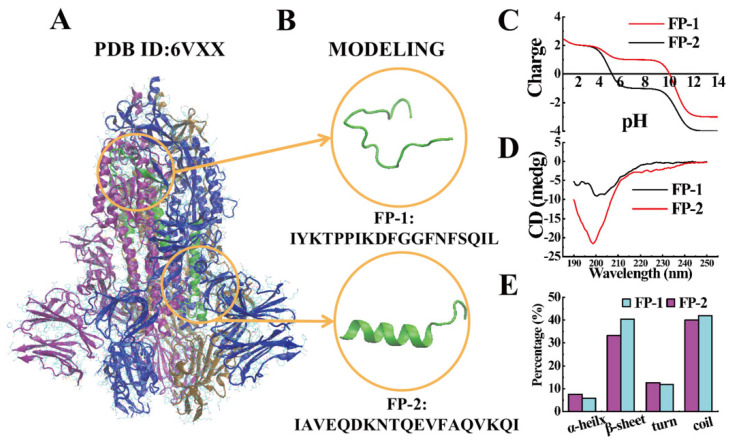
(**A**) Cryon-EM structure of SARS-CoV-2 S protein (PDB: 6VXX) [32] with the highlighted functional peptides (green). Each monomer is drawn in a different color (purple, blue, tan). (**B**) The SARS-CoV-2 functional-peptide structures were modeled using SARS-CoV-2 S protein. FP-1 adopts random-coil secondary conformation, and FP-2 adopts α-helical secondary conformation. (**C**) The plot of charge vs. pH of FP-1 and FP-2; ISO, isoelectric point. (**D**) CD of the secondary structure of FP-1 and FP-2 (66 µM) in solution. (**E**) The contents of secondary structures of FP-1 and FP-2 (66 µM).

In the experiment, the two functional peptides, FP-1 and FP-2 (Figure 1A,B) were selected and explored the basic charge property, and the opposite charge property was displayed in the plot of peptide charge vs. pH ranging from 0 to 14 (Figure 1C). The plot of peptide charge vs. pH showed the basic property of FP-1 and FP-2 with the pH ranging from 0 to 14 based on the following formula:

(1) $$Z=\sum_{i}Ni\frac{10^{pK_{ai}}}{10^{pH}+10^{pK_{ai}}}-\sum_{j}Nj\frac{10^{pH}}{10^{pH}+10^{pH_{aj}}}$$

*N_i_* is the number of the N-terminal and side chains of histidine, arginine, and lysine. *pK_ai_* is the *pK_a_* value of the N-terminal and side chains of histidine, arginine, and lysine. *N_j_* is the number of the C-terminal and side chains of aspartic acid, glutamic acid, cysteine, and tyrosine amino acids. *pK_aj_* is the *pK_a_* value of the C-terminal and side chains of aspartic acid, glutamic acid, cysteine, and tyrosine amino acids [29,30]. FP-1 showed the positive charge, while FP-2 displayed the negative charge at pH 7. However, the two functional peptides displayed the similar secondary structures dominated by random coil at pH 7, explored by circular dichroism (CD) spectra (Figure 1D). Furthermore, the content of secondary structure analysis of the functional peptides performed using the Dichro-Web online software [31] presented that a small amount of α-helix and β-sheet conformation still existed and the content of α-helix in FP-2 was slightly higher than that in FP-1 (Figure 1E).

**Figure 2:**
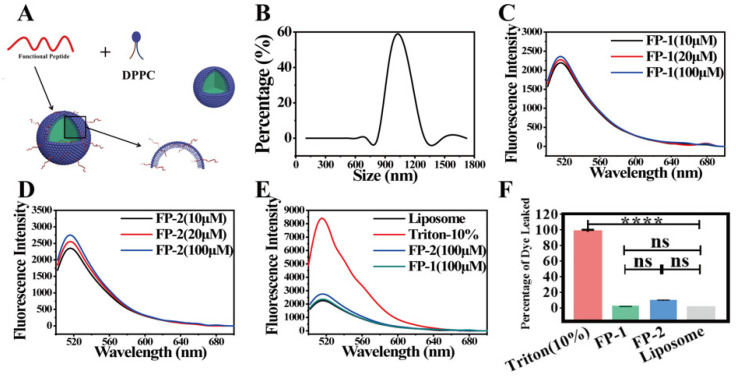
The interaction between the functional peptides of SARS-CoV-2 and DPPC liposomes. (**A**) Schematic diagram of the functional peptides interacting with DPPC liposomes. (**B**) Particle-size distribution of DPPC liposomes. (**C**,**D**) Dye-release assay of calcein-encapsulated DPPC liposomes induced by FP-1 and FP-2 (10 μΜ, 20 μΜ, 100 μΜ). (**E**) Dye-release assay of calcein-encapsulated DPPC liposomes induced by FP-1 and FP-2 (100 μΜ). Triton (10%) is the positive control. PBS is the negative control. (**F**) Percentage of dye leakage of each tested group. One-way analysis of variance (ANOVA) with Bonferroni’s correction, not significant (n.s.), **** *p* < 0.0001, error bars are standard deviation, s.d.

Subsequently, we prepared the DPPC liposomes containing calcein to mimic neutral mammalian cell membrane, and the average particle size of DPPC liposomes was 1 µm (Figure 2B). As depicted in Figure 2A, the functional peptides interacted with DPPC liposomes, causing few liposomes to aggregate and break (Figure S1). Furthermore, a classical dye-release assay was used to examine the ability of peptide to bind and disrupt the lipid membrane. The molar concentration ratios of peptide vs. lipid (P: L) were set as 1:100, 1:50, and 1:10, and the amounts of peptides were added to the calcein-contained DPPC liposomes according to the ratio above. Both peptides exhibited the weak capability of disrupting the liposome, and a very low amount of calcein was released from the liposome after the treatment of two functional peptides, compared to 10% Triton X-100 (Figure 2C–F). Among the three ratios of peptide vs. lipid, the functional peptides slightly induced the calcein release from liposome at the high ratio of peptide vs. lipid (1:10), which could be ignored compared to the case of 10% Triton X-100. Therefore, it is concluded that the functional peptides could bind to the liposome but rarely disrupt the membrane [33].

**Figure 3:**
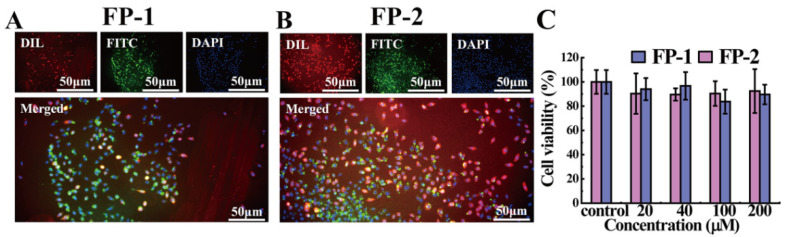
The interaction between the functional peptides of SARS-CoV-2 and the mouse fibroblast cell line (L929). (**A**,**B**) Fluorescence images of the mouse fibroblast cell line (L929)) treated with FP-1 and FP-2. Size: 50 µm. (**C**) Cell viability of the mouse fibroblast cell line (L929) treated by the functional peptides with different concentrations, and error bars denote standard deviation (s.d.).

Furthermore, we explored the binding activity of the functional peptides with the mammalian cell, the mouse fibroblast cell line (L929). It was found that the functional peptides modified with Fluorescein-5-isothiocyanate (FITC) showing green fluorescence can bind to the cell membrane, and the functional peptides localize the cell membrane and facilitate the whole cell to exhibit the green fluorescence, which matches the cell morphology very well in Figure 3A,B and Figure S2 (i.e., circled round cells and long cells, etc.) with relative uniform green fluorescence. It is demonstrated that the functional peptides are able to bind to the cell membrane. The cell viability of the mouse fibroblast cell line (L929) further proved that the functional peptides displayed no appreciable toxicity to the cell even at the concentration of 200 µM (Figure 3C), which implied that the functional peptides were not disrupting the cells.

In theoretical simulation, we further investigated the mechanistic insight of the functional peptides of SARS-CoV-2 binding lipid membrane. Four lipid membranes (DMPC, DPPC and POPC and POPG, well existing in the cell membrane) were selected as the cell-membrane models (the PC membrane would represent the mammalian cell membrane, and the POPG membrane would represent in the bacterial cell membrane) and the interactions between the functional peptides and lipid membrane were examined by molecular dynamics simulations.

**Figure 4:**
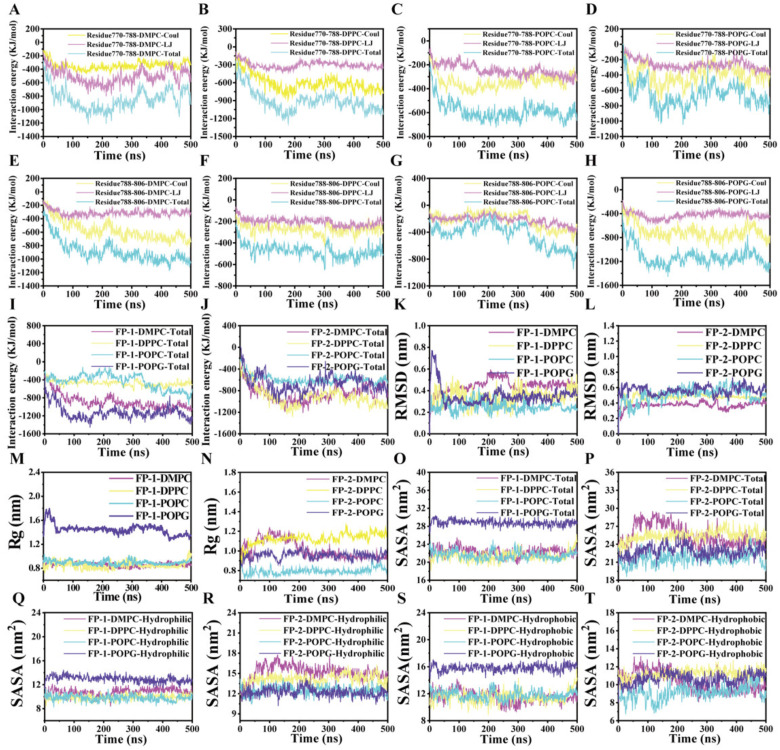
The data analysis during the whole simulation. (**A**–**H**) The time evolution of the interaction energy between these two functional peptides and the four lipid membranes, in terms of the electrostatic components (Coulomb) and van der Waals (LJ). (**I**,**J**) The time evolution of the total interaction energy for these two functional peptides in four lipid membranes during the whole simulation. (**K**,**L**) The time evolution of the RMSD for these two functional peptides in four lipid membranes during the whole simulation. (**M**,**N**) The time evolution of the Rg for the functional peptides in four lipid membranes during the whole simulation. (**O**,**P**) The time evolution of the SASA for these two functional peptides in four lipid membranes during the whole simulation. (**Q**–**T**) The time evolution of the SASA for these two functional peptides in four lipid membranes. The decomposition of the total SASA is the hydrophobic areas and the hydrophilic areas.

A comparison of the peptide’s structural drift in the simulations provides information regarding its conformational stability and overall conformational motion on the timescale of the simulations. The structural drift was evaluated by calculating the root-mean-square deviation (RMSD) of the backbone for the functional peptides during the whole simulation. The radius of gyration (Rg) can give insight into the general properties and behaviors of the functional peptides in lipid bilayer models, such as the degree of compactness. The solvent-accessible surface area (SASA) is another parameter that is used to evaluate the state of peptides and also is considered as an indicator of the accessibility of the surrounding solvent to a molecule. During the simulation process, the functional peptides spontaneously approached and contacted the lipid membrane, and then bound with the lipid membrane. As shown in Figure 4, the interaction energy gradually increased between the functional peptides and lipid membrane, and then reached a stable state. It can be seen from the curves of RMSD, Rg, and SASA that the system gradually tended to an equilibrium phase, which verified the reliability of the simulation results [34].

To further evaluate the effect of the functional peptides on the fluidity and affinity of lipid bilayer, the lateral diffusion coefficients of lipid molecules were calculated from the mean-square displacement (MSD), in the case of the functional-peptide binding with the lipid membrane during the whole simulation. We could calculate the D_mem_, D_mem+functional peptide_, and D_mem+functional peptide_/D_mem_, which represent the fluidity of the membrane and membrane bind by the functional peptides, as well as the affinity between the peptide and membrane. It was found that D_mem_ and D_mem+functional peptide_ displayed dramatically differently in all eight systems during the whole simulation, indicating the effect of the functional peptides on the diffusion and fluidity of the lipid membrane. The ratio of D_mem+functional peptide/_D_mem_ is less than 1, implying the confined effect of the functional peptides on the lipid-membrane diffusion, and also suggesting good affinity between the functional peptides and lipid membrane (Table 1). Based on the analysis from Table 1, FP-1 exhibited the best affinity with POPG membrane, while FP-2 displayed the best affinity with the DPPC membrane during the eight simulation systems with the relative lowest ratios.

To analyze the ensemble of the functional-peptide membrane-bound configurations captured in the eight performed simulations, clustering was performed using the binding modes of individual peptide chains with the membrane (Figure S3) to identify stable membrane binding. The most dominant cluster (cluster number 1) of each simulation was found, which represented the equilibrium phases in the final time quantum (t = 475–500 ns), and thus we selected the functional-peptide membrane-bound configuration at the final time (t = 500 ns) for subsequent free-energy predictions.

**Figure 5:**
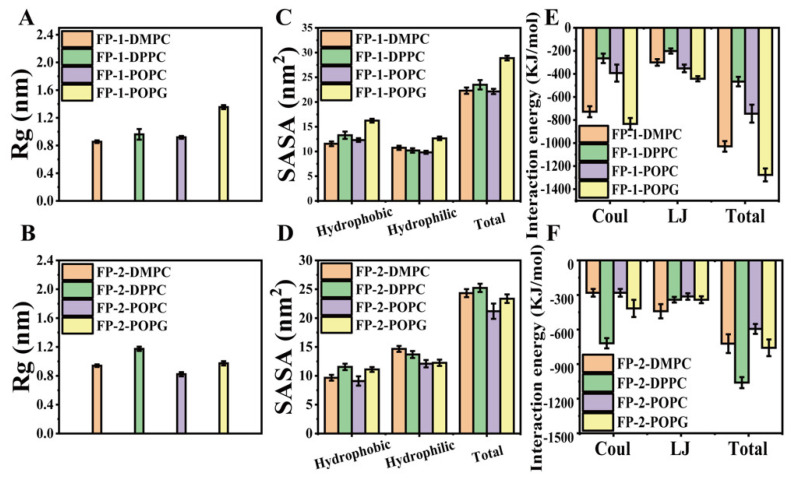
Analysis of the simulation process between the functional peptides and the lipid membrane at equilibrium performed in the last 25 ns of the simulation (t = 475–500 ns). (**A**,**B**) The radius of gyration average values for the functional peptides in different lipid membranes. (**C**,**D**) The solvent accessible surface area average amounts for functional peptides in different lipid membranes. (**E**,**F**) Decomposition of the total interaction (kJ/mol) between the functional peptides and different lipid membranes, in terms of the electrostatic components (Coulomb) and van der Waals (LJ). Error bars denote standard deviation (s.d.).

In the equilibrium phase, FP-1 presented the largest Rg in the POPG membrane, which was approximately 1.4 nm, while FP-2 showed the largest Rg in the DPPC membrane, which was approximately 1.2 nm, indicating that the functional peptides were fluffy (Figure 5A,B). Hence, the SASA is calculated to evaluate the hydrophilicity and hydrophobicity of the functional peptides in DMPC, DPPC, POPC, and POPG lipid membrane. In general, it is suggested that the hydrophobic area was larger than the hydrophilic area in the case of FP-1 (Figure 5C), on the contrary, the hydrophilic area was larger than the hydrophobic area in general for FP-2 (Figure 5D). Moreover, FP-1 displayed the largest hydrophobic area in the POPG membrane, which was approximately 17 nm^2^, while FP-2 exhibited the largest hydrophilic area in the DMPC membrane, which was approximately 12 nm^2^, compared to lipids, respectively. The general property of amphiphilicity of the functional peptides was explored in membrane binding, which should be able to bind the exposed residues in solution form.

Moreover, the energy analysis was performed to confirm and quantify the interaction between the functional peptides and the four lipid membranes. Calculations have been performed with the g_energy program of the GROMACS suite. The total interaction energy is actually contributed from the electrostatic interaction (Coulomb) and van der Waals (LJ) contributions. However, FP-1 showed the maximum interaction energy with the POPG membrane (−1218 kJ/mol), and the second one with the DMPC membrane (−990 kJ/mol), compared to the ones with the other two lipid bilayers (Figure 5E,F). It was clearly observed that the total interaction energy between FP-2 and the three lipid membranes (DMPC, POPC, and POPG) were the same order of magnitude, indicating a slight difference. However, in the case of DPPC membrane, the total interaction between FP-2 and the DPPC membrane is determined to be −1122 kJ/mol, implying good stability of binding between peptide and membrane. Small-molecule drugs generally enter cells in the body through a difference in concentration. It is difficult for the drug itself to enter cells, which will be affected by various factors such as the effect of the cholesterol of cell membranes on cellular uptake of the cancer drugs pirarubicin and ellipticine [35]. Because of the binding effect of the functional peptide with the membrane, we envisioned that the functional peptide could deliver small drug molecules into the cell membrane, providing aid for the treatment of SARS-CoV-2. For example, the cell-penetrating peptide (NP1) is presented to encapsulate and deliver an anticancer drug ellipticine (EPT) into two model cells: non-small-cell lung carcinoma and Chinese hamster ovary cells [36].

To further probe the energetics of the functional peptides and the lipid-membrane interaction, the atomic-resolution potential of mean force (PMF) profiles for the functional peptides and lipid membrane were calculated for each system. The final atomistic coordinates of all-atom equilibration simulations were used as the initial configurations for umbrella sampling. In all systems, those coordinates lay within the most populated cluster, and the PMF representing disassociated energies of FP-1 with POPG membrane and FP-2 with DPPC membrane were calculated to be approximately 48.4 kcal·mol^−1^ and 44.2 kcal·mol^−1^, respectively (Table 1 and Figure S4). It showed the maximum disassociated energy in each lipid membrane, which was consistent with the results obtained above that FP-1 could be apt to bind the POPG membrane and FP-2 would be inclined to bind the DPPC membrane. The trends of PMF variation in other lipid membranes are also consistent with the conclusion obtained above [37,38].

**Figure 6:**
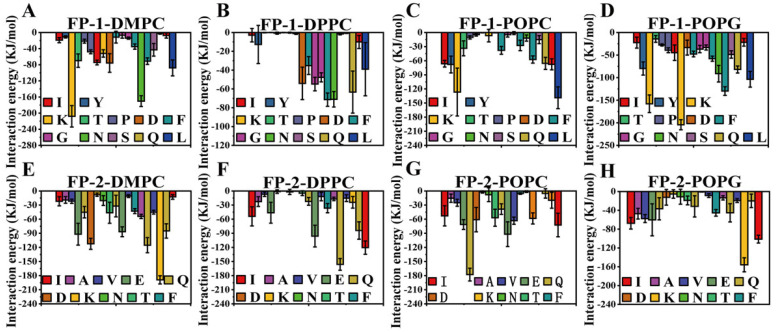
Analysis of the simulation process between the functional peptides and the lipid membrane at equilibrium was performed in the last 25 ns of the simulation (t = 475–500 ns). (**A**–**H**) The interaction-energy average values between each amino acid. Error bars denote standard deviation (s.d.).

Subsequently, we explored the key amino acids of the functional peptides binding with the lipid membrane by evaluating the interaction energy between amino acids and the lipid membrane at an equilibrium phase (Figure 6). In the case of FP-1, Lys (K), Ile (I), Asn (N) and Gln (Q) played a significant role in neutral phospholipid-membrane binding, and only Lys (K) exhibited the excellent ability to bind negatively charged lipid membrane, the maximum interaction energy between the Lys (K) of FP-1 and POPG membrane was determined to be −200 kJ/mol. In another case of FP-2, Lys (K), Ile (I), and Gln (Q) plays the key role in the neutral lipid-membrane-binding process, while Lys (K) and Ile (I) are key ones in negatively charged lipid-membrane binding; the maximum interaction energy between the Lys (K) of FP-2 and POPG membrane was determined to be −180 kJ/mol. Most of these amino acids are electrically charged and hydrophobic, which could be a feature of the functional peptides.

**Figure 7:**
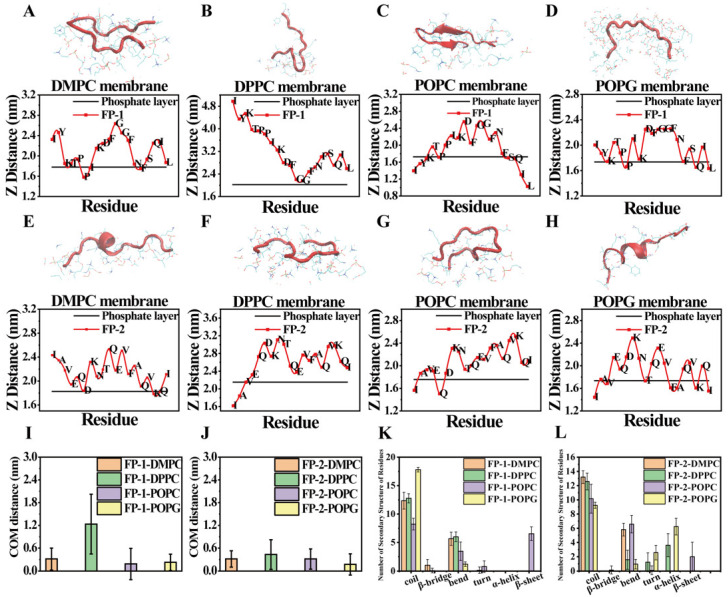
Adsorption of two functional-peptide residues to four lipid membranes. (**A**–**H**) The binding-configuration plots of the two functional peptides adsorbed onto the lipid bilayer (DMPC, DPPC, POPC, POPG). The two functional peptides are shown in red cartoon model; lipids are represented by blue lines. Plots showed the COM distance at Z direction of each amino acid with respect to the phosphate layer (black) of the bilayer (t = 500 ns). (**I**,**J**) The average COM distance between each amino-acid residue and the phosphate groups in every membrane system (t = 500 ns). Error bars denote standard deviation (s.d.). (**K**,**L**) The contents of secondary structures of the two functional peptides in four lipid membranes (t = 475–500 ns). Error bars denote standard deviation (s.d.).

Furthermore, among the eight performed membrane-binding simulations, all stable binding events were observed and recorded (Figure 7). Spontaneous diffusion and membrane binding of the functional peptides were examined and evaluated by the position of the center of mass (COM) of individual membrane-bound residues of the functional peptides in each simulation at 500 ns. The Z component of the COM was tracked with respect to the phosphate layer (the Z position of all the phosphate groups) of each leaflet (black lines, respectively, in Figure 7A–H). From the plots of residue–lipid interactions, we found two membrane-binding states, the whole functional peptide (Figure 7A,B,D–H) and the C-terminus of the functional peptide (Figure 7C) interacting with the lipid membrane. We calculated the average COM distance between the functional peptides and the phosphate groups, and we concluded that the average COM distance between FP-1 and DPPC membrane was 1.23 nm, and the ones of other systems were all fluctuating around 0.3 nm (Figure 7I,J). It represented the states of the functional peptides interacting with a lipid bilayer. The secondary structures of the functional peptides are generally retained in the random coil during the membrane binding at t = 475–500 ns, but there is still a slight difference in eight cases. For FP-1, the random coil was largely retained, especially in the case of POPG membrane binding. In the case of FP-2, the content of the random coil decreased in DMPC, DPPC, POPC, and POPG, respectively, while the content of alpha-helix increased slightly. Calculations were performed with the g_do_dsssp program of the GROMACS suite (Figure 7K,L).

**Figure 8:**
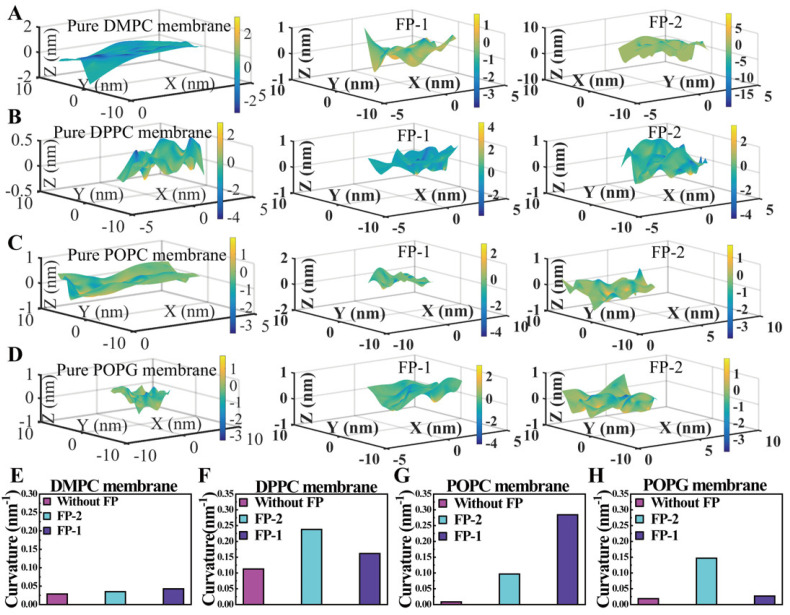
Analyses of the curvature of the lipid membrane at the end of simulation (t = 500 ns). (**A**–**D**) Three-dimensional surface of the upper layer of lipid membranes without and with two functional peptides. (**E**–**H**) Curvature of the upper layer of lipid membranes without and with functional peptides.

Finally, the curvature of the lipid membrane could also give the contribution of evaluating the activity of the functional-peptide-binding membrane. In general, the functional peptides exhibited different local indentation effects on the lipid membranes (Figure 8A–D) [22,39], due to the distinct binding activities of the functional peptides on four lipid membranes (Figure 8E–H). Qualitatively, the functional peptides binding on the membrane could increase the curvature of the lipid membrane, which might be ascribed to the pressure of the upper leaflet of the lipid membrane induced by the binding of the functional peptides, and thereby reducing the mechanical tension of lipid membrane [40]. 

## 4. Conclusions

In this work, through the combination of experiment and simulation, we have explored the interaction of the functional peptides from SARS-CoV-2 with the cell membrane. The functional peptides could bind to the cell with little disruption, indicating the nontoxicity of peptides. In the theoretical simulation, the two functional peptides, FP-1 and FP-2, exhibit different membrane-contacting ability, and in the case of FP-1, peptide prefers to contact with the POPG membrane, whereas the DPPC membrane is preferred by FP-2. The specific amino acids (i.e., Lys, Ile, Glu, Asn, Gln, etc.) with charge or hydrophobic residue play a significant role during the functional peptides binding to the membrane. The results obtained in this work verified that functional peptides could bind to the membrane and provide a possible theoretical insight into the interaction between peptides and the cell membrane, and also might pave the way for development of new materials based on peptides with membrane-binding activity, and the peptides would be further enabled as peptide adjuvants in order to help deliver the cancer drug into tumor cells for efficient tumor therapy.

## Figures and Tables

**Table 1:** Measured dynamic, structural, and energetic properties from atomic-resolution simulations of the functional peptides ^a^.

Functional Peptide	Lipid Bilayer	D_MDmem_ (μm^2^ s^−1^)	D_MDmem+functional peptide_ (μm^2^·s^−1^)	D_MDmem+functional peptide_/D_MDmem_	No. of Clusters Obtained	PMF (kcal·mol^−1^)
FP-1	DMPC	1.75 ± 0.047	1.4 ± 0.047	0.8	2	22.515 ± 0.531
	DPPC	4.1 ± 0.029	3.82 ± 0.029	0.932	4	28.369 ± 1.10
	POPC	1.3 ± 0.019	1.07 ± 0.019	0.826	2	20.030 ± 1.396
	POPG	2.1 ± 0.066	1.26 ± 0.066	0.602	4	48.458 ± 0.922
FP-2	DMPC	1.75 ± 0.047	1.225 ± 0.042	0.7	9	41.022 ± 0.578
	DPPC	4.1 ± 0.029	1.425 ± 0.274	0.347	3	44.260 ± 0.902
	POPC	1.3 ± 0.0019	1.0 ± 0.018	0.769	3	24.458 ± 1.266
	POPG	2.1 ± 0.0066	1.7 ± 0.075	0.818	4	28.737 ± 0.729

^a^ The lateral diffusion coefficients in the presence (Dmem+functional peptide) or absence (Dmem) of peptide were extracted from a least-squares fit of the most linear 500 ns window of the mean-square displacement (MSD) plot with respect to simulation time. Dmem+functional peptide represents the lateral diffusion coefficient only for the leaflet to which the peptide is bound, while Dmem represents the lateral diffusion coefficient for both leaflets. Errors in diffusion coefficients correspond to differences obtained from fits over the two halves of each 500 ns window. The functional peptide and lipid-membrane-binding energy (PMF) was calculated as the difference between the maximum plateau region in solution and the minimum within the lipid-bilayer environment (observed around the membrane headgroup interfacial region). Clustering was performed using the GROMOS method for C-alpha atoms, with an RMSD cutoff of 0.45 nm.

## Data Availability

Not applicable.

## Supplementary Materials

The following supporting information can be downloaded at: https://www.mdpi.com/article/10.3390/cells11111738/s1, Figure S1: The interaction between the functional peptides (FP-1-FITC) and DPPC liposomes. Figure S2: The interaction between the functional peptides (FP-1-FITC and FP-2-FITC) and the mouse fibroblast cell line (L929). Figure S3: The data analysis during the whole simulation. Figure S4: Time-dependent cluster occurrence for each functional peptide in the DMPC, DPPC, POPC, or POPG lipid membranes. Figure S5: The PMF between these functional peptides and the four lipid membranes associated with the distance (ζ) are indicated.
