# Supplementary material for: Functional Peptides from SARS-CoV-2 Binding with Cell Membrane: From Molecular Dynamics Simulations to Cell Demonstration
Source: Cells. 2022 May 25;11(11):1738. doi: 10.3390/cells11111738 (PMC9179371; doi:10.3390/cells11111738)
Supplement: Supplementary file 1 [file cells-11-01738-s001.zip › cells-1713300-supplementary.pdf]

cells

Supplemental information

Functional Peptides from SARS-CoV-2 Binding with Cell Membrane: From Molecular Dynamics Simulations to Cell Demonstration

Yun Hao #, Rongrong Wu #, Fenghua Wang, Liwei Zhang, Zengkai Wang, Xiaolu Song, Lei Liu*.

Supporting Figures

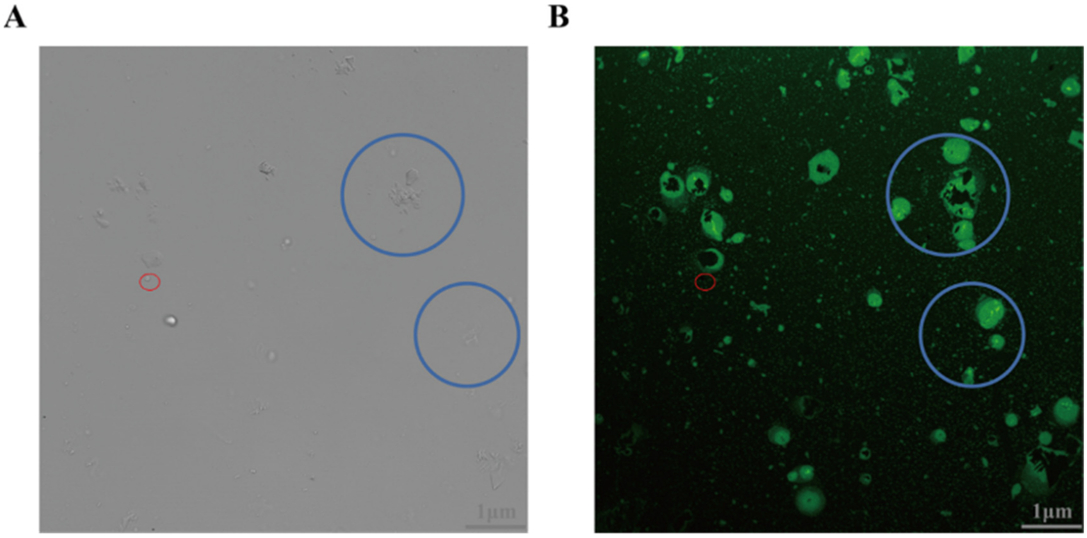

Figure S1. The interaction between the functional peptides (FP-1-FITC) and DPPC liposomes.

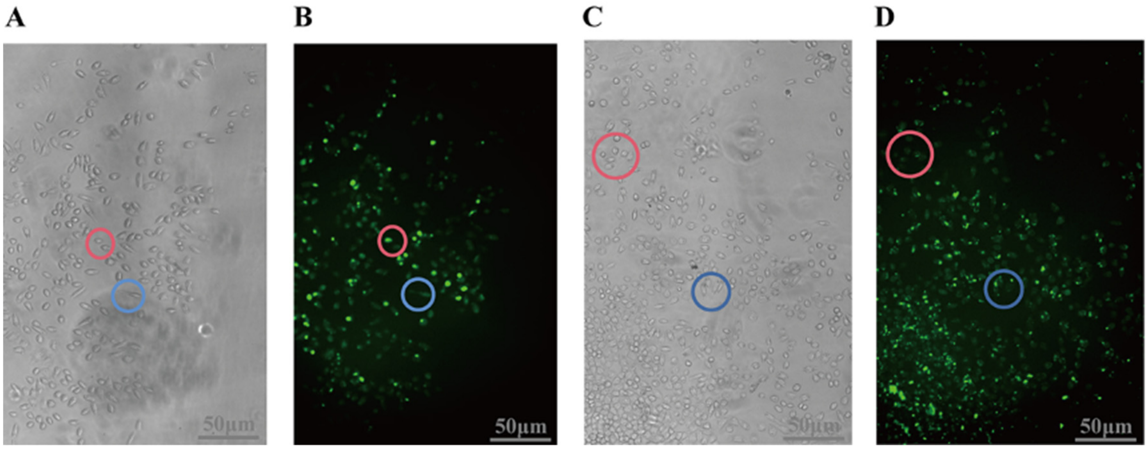

Figure S2. The interaction between the functional peptides (FP-1-FITC and FP-2-FITC) and the mouse fibroblasts (L929).

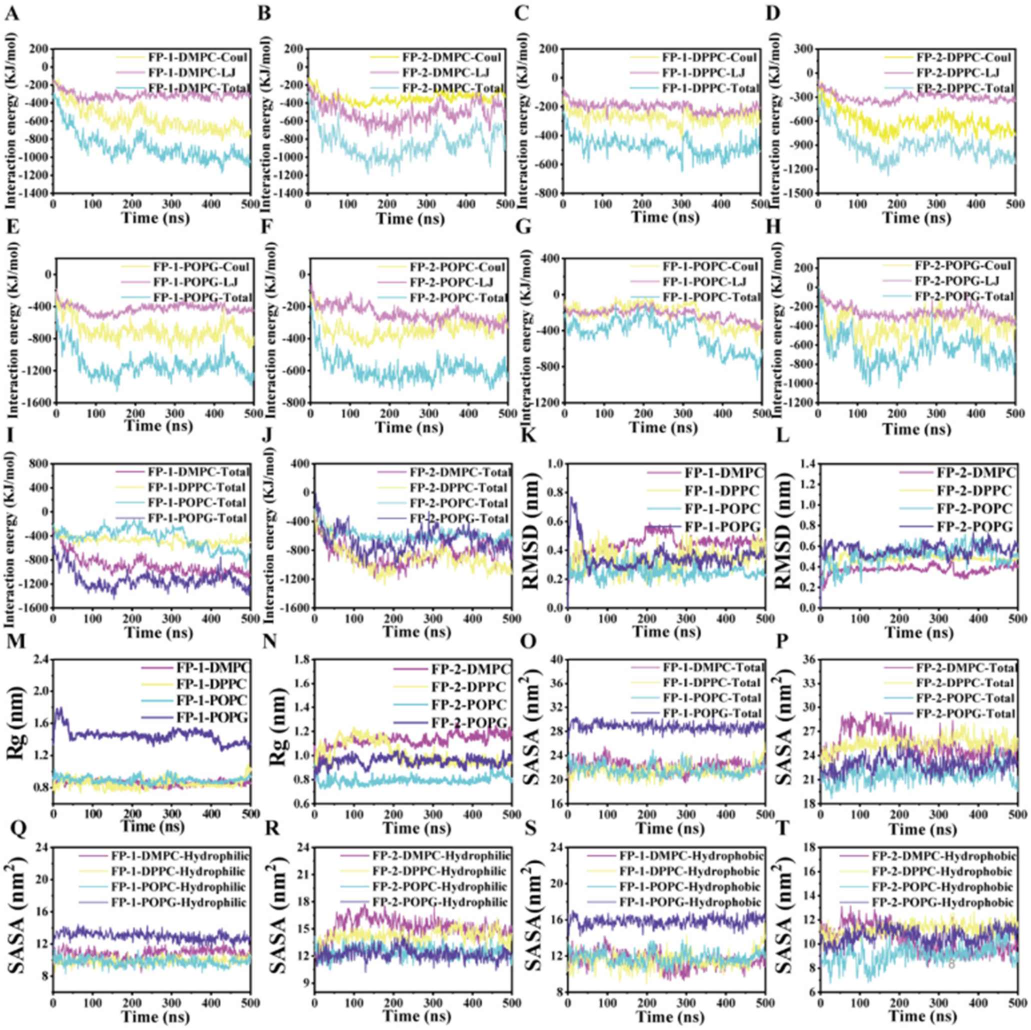

Figure S3. The data analysis during the whole simulation. (A-H) The time evolution of the interaction energy between these two functional peptides and the four lipid membranes, in terms of the electrostatic components (Coulomb) and van der Waals (LJ). (I, J) The time evolution of the interaction energy for these two functional peptides in four lipid membranes during the whole simulation. (K, L) The time evolution of the RMSD for these two functional peptides in four lipid membranes during the whole simulation. (M, N) The time evolution of the Rg for the functional peptides in four lipid membranes during the whole simulation. (O, P) The time evolution of the SASA for these two functional peptides in four lipid membranes during the whole simulation. (Q-T) The time evolution of the SASA for these two functional peptides in four lipid membranes. The decomposition of the total SASA is the Hydrophobic areas and the Hydrophilic areas.

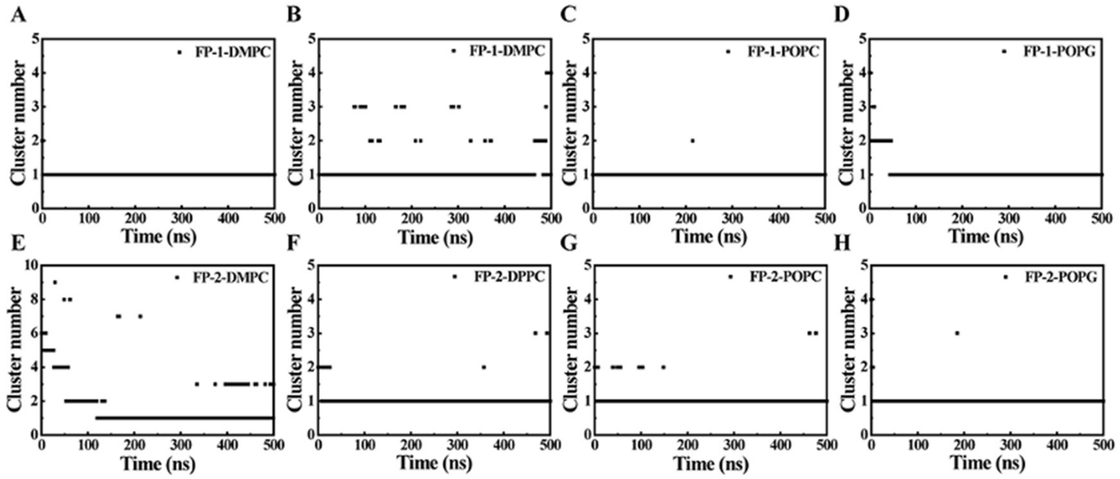

Figure S4. Time dependent cluster occurrence for each functional peptide in the DMPC, DPPC, POPC or POPG lipid membranes. The cluster number occurrence over simulation time is shown, ordered by dominance. The final coordinates of each simulation were found within the most dominant cluster (cluster number 1) and were thus selected for subsequent free energy predictions. Clustering was performed using the Gromos method for C-alpha atoms, with an RMSD cut-off of 0.45 nm.

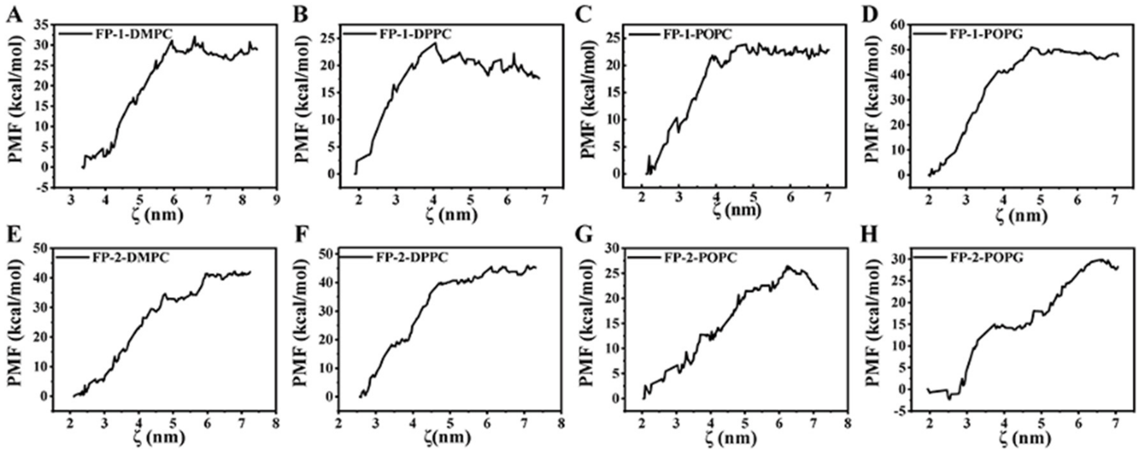

Figure S5. The PMF between these functional peptides and the four lipid membranes associated with the distance (ζ) are indicated.
